# The rise of carbapenem resistance in Europe: just the tip of the iceberg?

**DOI:** 10.1186/2047-2994-2-6

**Published:** 2013-02-14

**Authors:** Anna-Pelagia Magiorakos, Carl Suetens, Dominique L Monnet, Carlo Gagliotti, Ole E Heuer

**Affiliations:** 1 European Centre for Disease Prevention and Control, Tomtebodavägen 11A, Stockholm SE-171 83, Sweden; 2 Agenzia Sanitaria e Sociale Regionale Emilia-Romagna, Bologna, Italy

**Keywords:** Carbapenems, Antimicrobial drug resistance, Gram-negative bacteria, *Klebsiella* infections, Europe, β-lactamases

## Abstract

The European Antimicrobial Resistance Surveillance Network (EARS-Net) collects data on carbapenem resistance from invasive bacterial infections. Increasing percentages of carbapenem resistance in *K*. *pneumoniae* isolates were reported from progressively more countries in Europe between 2005 and 2010. A trend analysis showed increasing trends for Greece, Cyprus, Hungary and Italy (p < 0.01). EARS-Net collects data on invasive bacterial isolates, which likely correspond to a fraction of the total number of infections. Increasing reports of community cases suggest that dissemination of carbapenem-resistant *K*. *pneumoniae* has penetrated into the community. Good surveillance and infection control measures are urgently needed to contain this spread.

## Background

European Antimicrobial Resistance Surveillance Network (EARS-Net) data originate from clinical microbiological laboratories across Europe, reporting antimicrobial susceptibility results for seven invasive bacterial pathogens. These include two of the *Enterobacteriaceae*, *Klebsiella pneumoniae* (*K*. *pneumoniae*), a common cause of healthcare-associated infections, and *Escherichia coli* (*E*. *coli*), a frequent cause of community-acquired urinary tract infections, which is widely disseminated in the environment. In general, carbapenem resistance in *Enterobacteriaceae* can be conferred by the presence of various mechanisms of resistance, including the carbapenemases, which are β-lactamases that can hydrolyse most β-lactams, including the carbapenems. Good prevalence data are not always available for carbapenemase-producing *Enterobacteriaceae* (CPE) and since carbapenem-resistant *K*. *pneumoniae* isolates are frequently found to be carbapenemase-producing, carbapenem resistance is frequently used a surrogate marker for the presence of carbapenemases.

Infections with CPE were first reported in Europe as imported cases or outbreaks in healthcare systems from endemic countries within Europe and beyond [1,2]. The epidemiology of CPE in Europe is now changing and an increasing number of community-associated, autochthonous cases are reported [1,3]. Interestingly enough, recent publications describe the detection of CPE from environmental sources, *i*.*e*. KPC-producing *E*. *coli* and OXA-48-producing *Serratia marcescens* from environmental water samples in Portugal [4] and Morocco [5], respectively, suggesting that sources of CPE go beyond the hospital, into the community and environment.

Data from EARS-Net showed increasing percentages of carbapenem resistance in *K*. *pneumoniae* isolates between 2005 and 2010 from progressively more countries in Europe. A trend analysis in the EARS-Net Annual Report 2011 showed an increasing trend for Europe, overall [6]. Some countries were not included in this analysis because their laboratories did not report continuously during 2005–2010. Among the remaining countries one country, Greece, contributed 27% of the total *K*. *pneumoniae* isolates and 98% of carbapenem-resistant *K*. *pneumoniae* isolates and drove most of the observed increasing trend. Our study aimed to look at and separately analyse the trends for the individual countries reporting an increase of carbapenem resistance in *K*. *pneumoniae* isolates to EARS-Net from 2005 to 2010.

## Methods

Susceptibility testing results of invasive, carbapenem- resistant *K*. *pneumoniae* isolates (not including intermediately susceptible), were extracted from the EARS-Net database for 2005–2010. Laboratories included in the analysis were those that had reported antimicrobial susceptibility results for *K*. *pneumoniae* to EARS-Net for at least three years during 2005–2010. Trend analyses, using number of isolates and not percentages, were performed using the Cochran-Armitage test for trend and confirmed by Poisson regression.

## Results

**Figure 1:**
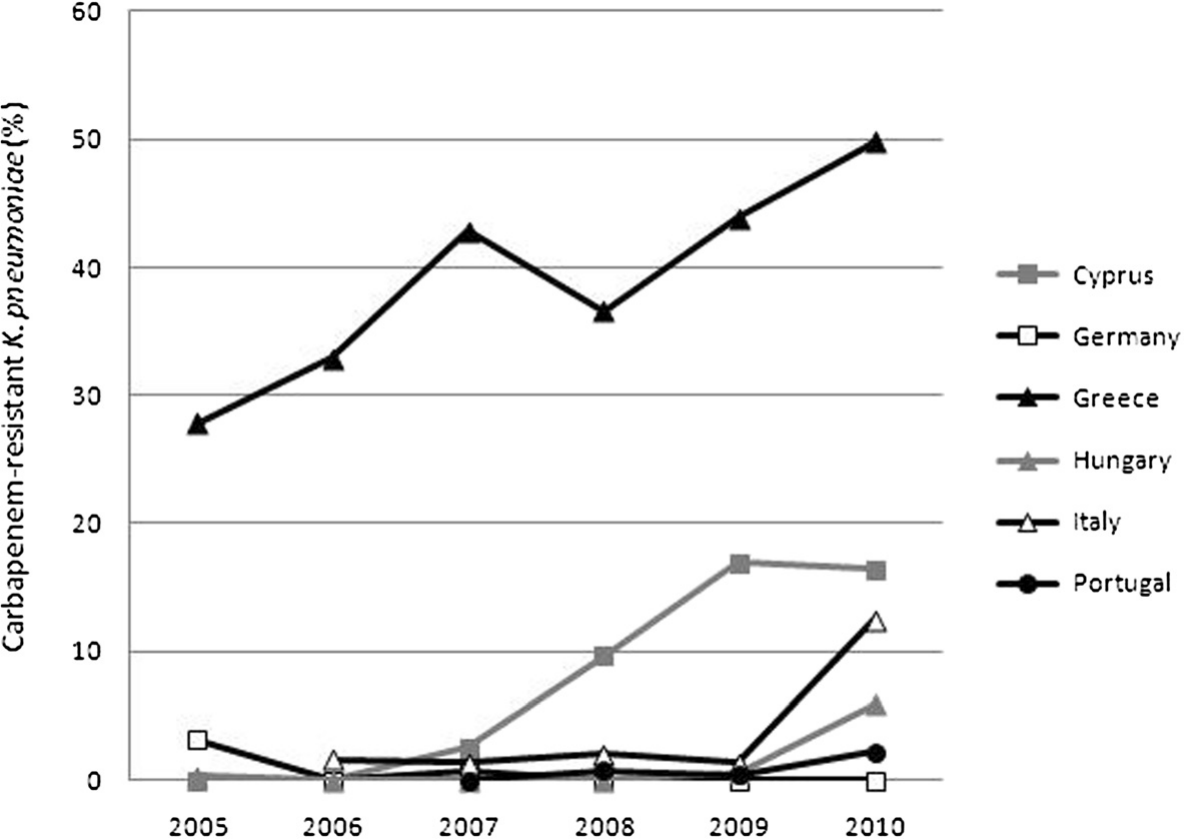
**Carbapenem**-**resistant *K***. ***pneumoniae *trends from the European Antimicrobial Resistance Surveillance Network (EARS-Net), 2005 to 2010. **Percentages and trends of invasive carbapenem-resistant *K*. *pneumoniae *isolates from European countries reporting to the European Antimicrobial Resistance Surveillance Network (EARS-Net) from 2005 to 2010. The 6 countries shown in this figure are those whose laboratories reported carbapenem susceptibility data in *K*. *pneumoniae *isolates for at least three years during 2005–2010, had a percentage of 1% or higher for any of these years and for which a significantly increasing or decreasing trend was observed. The number of countries with a percentage resistance of 1% or higher, increased from 2 in 2005 (Greece, 27.8%; Germany, 3.1%) to 5 in 2010 (Greece, 49.8%; Cyprus, 16.4%; Italy, 12.5%; Hungary, 5.9%; Portugal, 2.2%). Significant increasing trends were observed for Greece, Cyprus, Hungary and Italy (p < 0.01). Germany, which did not report any carbapenem-resistant *K*. *pneumoniae *isolate in 2010, showed a decreasing trend (p < 0.01).

Eighteen countries reported at least one carbapenem-resistant *K*. *pneumoniae* isolate in the six-year period and of these countries, six showed an increasing or decreasing trend. The number of countries with ≥1% carbapenem resistance amongst invasive *K*. *pneumoniae* isolates increased from 2, in 2005 (Greece, 27.8%; Germany, 3.1%) to 5 in 2010 (Greece, 49.8%; Cyprus, 16.4%; Italy, 12.5%; Hungary, 5.9%; Portugal, 2.2%). Significant increasing trends were observed for Greece, Cyprus, Hungary and Italy (p < 0.01). Germany, which did not report any carbapenem-resistant *K*. *pneumoniae* isolate in 2010, showed a decreasing trend (p < 0.01) (Figure 1).

## Discussion

Increasing trends of resistance to carbapenems in *K*. *pneumoniae* isolates in Europe, even if currently observed only in a few countries, are worrisome because carbapenems are last-line antibiotics. Additionally, when carbapenem resistance is due to presence of carbapenemases, accumulation of other resistance traits to aminoglycosides and fluoroquinolones often render these CPE extensively drug-resistant (XDR) or pandrug-resistant (PDR), leaving few or no effective treatment options [1,7,8]. These bacteria disseminate rapidly within hospitals, following breaches in infection control measures. Infections with CPE are associated with high patient morbidity and mortality [9,10].

Our knowledge of the magnitude and geographical distribution of carbapenem- resistant *Enterobacteriaceae* (CRE) is incomplete and EARS-Net data likely represent only “the tip of the iceberg” for a number of reasons. EARS-Net collects only data on invasive bacterial isolates and since these bacteria can also cause non-invasive infections, EARS-Net data likely correspond to only a fraction of the total number of these. Supporting this, are recent reports of autochthonous and community cases of CPE [1,3], suggesting the dissemination and penetration of CPE beyond the hospital, into the community. Lastly, variations in practices for detection, surveillance, reporting, notification of CRE and CPE, may not allow all cases to be reported to EARS-Net.

Heightened concern for this public health threat has spurred the publication of systematic reviews, risk assessments [1] and guidance documents [1,11-13], which address the control and spread of CPE. These underscore that good surveillance and early warning systems at all levels, active screening of high-risk patients, notification of health authorities, strict implementation of targeted infection control measures in healthcare systems and the practice of prudent use of antimicrobials are key elements to halt the spread of CPE.

EARS-Net data and interactive database are available at:

http://ecdc.europa.eu/en/activities/surveillance/EARS-Net/database/Pages/database.aspx.

## Competing interests

The authors report no financial or other competing interests relevant to this manuscript.

## Authors’ contributions

AM and OH are responsible for the concept and writing the manuscript. CS and CG performed for the statistical analyses. DLM, CS and the EARS-Net coordination group critically revised the manuscript. The EARS-Net representatives of national participants are responsible for providing data. All authors read and approved the final manuscript.
